# Transactions N. Y. State Medical Association for 1884

**Published:** 1885-07

**Authors:** 


TRANSACTIONS OF THE NEW YORK STATE MEDICAL ASSOCIATION, for the year 1884, Vol. I. Edited for the Association by Austin Flint, Jr., M. D. New York: D. Appleton & Co., Publishers. Atlanta, Ga.: S. P. Richards & Son.

This is a handsome volume of 654 pages, containing the proceedings of the first annual session of this Association, together with the papers read and the discussions had thereon. It reflects credit alike upon the editor and publishers.

